# Characteristics and Kinetic Analysis of AQS Transformation and Microbial Goethite Reduction:Insight into “Redox mediator-Microbe-Iron oxide” Interaction Process

**DOI:** 10.1038/srep23718

**Published:** 2016-03-29

**Authors:** Weihuang Zhu, Mengran Shi, Dan Yu, Chongxuan Liu, Tinglin Huang, Fengchang Wu

**Affiliations:** 1 Key Laboratory of Northwest Water Resources, Environment and Ecology, Ministry of Education, Xi’an University of Architecture and Technology, Xi’an 710055, China; 2 Pacific Northwest National Laboratory, Richland, Washington 99352, United States; 3 State Key Laboratory of Environmental Criteria and Risk Assessment, Chinese Research Academy of Environmental Sciences, Beijing 100012, China

## Abstract

The characteristics and kinetics of redox transformation of a redox mediator, anthraquinone-2-sulfonate (AQS), during microbial goethite reduction by *Shewanella decolorationis* S12, a dissimilatory iron reduction bacterium (DIRB), were investigated to provide insights into “redox mediator-iron oxide” interaction in the presence of DIRB. Two pre-incubation reaction systems of the “strain S12- goethite” and the “strain S12-AQS” were used to investigate the dynamics of goethite reduction and AQS redox transformation. Results show that the concentrations of goethite and redox mediator, and the inoculation cell density all affect the characteristics of microbial goethite reduction, kinetic transformation between oxidized and reduced species of the redox mediator. Both abiotic and biotic reactions and their coupling regulate the kinetic process for “Quinone-Iron” interaction in the presence of DIRB. Our results provide some new insights into the characteristics and mechanisms of interaction among “quinone-DIRB- goethite” under biotic/abiotic driven.

The high abundance and the redox sensitivity of Fe(III) oxides in natural environment have led to the extensive research in their redox biogeochemical behaviors^1^. The anaerobic environments such as sediments, soils, sediment-water interface and aquifers commonly contain organic matters with sensitive chemical structures that can act as redox mediator. Redox mediators, such as humic substances containing quinone structures, which have the redox potentials to facilitate electron acceptance and donation, can mediate the extracellular electron transferring from a bacterium to an insoluble electron acceptor, such as iron oxides. Extensive researches have consequently been performed to investigate the role of redox mediators in enhancing the degree and extent of microbial iron reduction^2^,^3^,^4^,^5^,^6^,^7^,^8^,^9^,^10^.

Quinone, as a redox mediator, can be reduced to a corresponding hydroquinone by dissimilatory iron reduction bacterium (DIRB)^11^,^12^,^13^. The hydroquinone can be abiotically oxidized by transferring electrons to electron acceptors^14^, such as Fe(III) oxides including ferrihydrite^15^,^16^, lepidocrocite^9^,^15^, hematite^17^,^18^and goethite^19^. These previous studies generated good knowledge of how redox mediators such as quinoine interact with Fe(III) oxides. Most of these studies, however, focused on the abiotic redox reaction between the reduced form of a redox mediator and iron oxides. These studies have established abiotic kinetics to describe the redox transformation between redox mediators and iron oxides^20^. The kinetic knowledge and the established models have been conceptualized to describe microbial reduction of iron oxides mediated by redox mediators^21^,^22^,^23^,^24^.

However, research on the coupling biotic and abiotic interactions between reduced redox mediators and iron oxides in the presence of DIRB should be needed further investigation^25^. The reduced redox mediator can be re-generated in the presence of DIRB by reducing oxidized redox mediator to its corresponding reduced form, thus enhancing the reduction capacity and reaction duration. In addition, iron oxides can be directly reduced by DIRB that may affect abiotic reduction by the redox mediator. For example, the Fe(II) produced by the microbial reduction can be adsorbed on residual iron oxide surfaces, which can impact subsequent redox cycle of quinoine and iron reduction. Therefore, it is important to understand the behavior of “Quinone-Iron” interaction system in the presence of DIRB.

In our current study, the humic substance containing quinoid structure, anthraquinone-2-sulfonate(AQS), and goethite were selected as the model redox mediator and iron oxide respectively, with the aim at the investigation of the transformation between oxidized and reduced species of redox mediator during the process of microbial reduction of iron oxide. Two pre-incubation reaction systems termed subsequently as the “DIRB-iron oxide” and “DIRB-redox mediator”, were specifically selected to investigate the interactions among “quinone-DIRB-iron oxide” under biotic/abiotic driven. The temporal variations in concentrations of quinoine and different species of reductively produced ferrous iron were monitored to determine redox reactions.

The specific objects of current study were to identify the kinetic characteristics and elucidate the possible transformation pathways of redox mediator during the process of microbial goethite reduction. Results from this study give insights into the interaction process of “Quinone-Iron-DIRB” by two specifically designed pre-incubation reaction systems, which were previously unrecognized, in the transformation between oxidized and reduced species of the redox mediator under biotic/abiotic driven

## Results

### The characteristics of microbial goethite reduction in “strain S12-redox mediator” pre-incubation reaction systems

**Figure 1:**
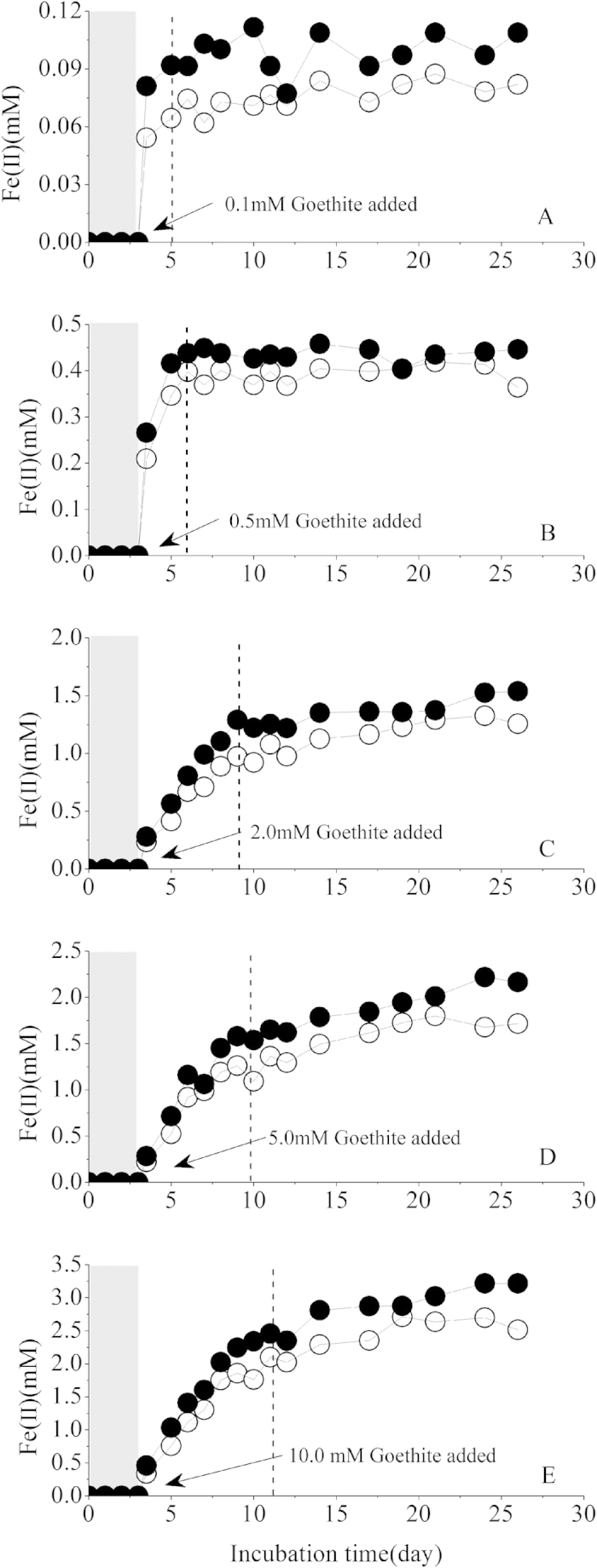
Concentrations of Fe(ΙΙ)_dis_ and Fe(ΙΙ)_tot_ in “strain S12- AQS” pre-incubation reaction systems added with different content of goethite after 3 days incubation. (Solid symbol: Fe(ΙΙ)_tot_, open symbol: Fe(ΙΙ)_dis_. Reaction systems components: AQS: 0.1 mM; goethite: 0.1 ~ 10.0 mM. The reaction systems containing 0.1 mM AQS inoculated with strain S12 at density of 2.5 × 10^8^ cells·ml^−1^, then after 3 days pre-incubation, different contents (0.1, 0.5, 2.0, 5.0 and 10.0 mM) of goethite were added to each pre-incubation reaction systems (**A–E**) respectively).

Both the concentrations of dissolved reductively produced ferrous iron(Fe(II)_dis_) and total reductively produced ferrous iron(Fe(II)_tot_) increased significantly (Fig. 1) in the “strain S12-redox mediator” pre-incubated reactors spiked with goethite of different concentrations. The reductively production of ferrous iron was attributed to the iron reducing bacterial respiration and the abiotic reduction of goethite by the reduced form of the redox mediator (AQS_red_). AQS_red_ was formed during the pre-incubation duration (3 days) (Fig. 1A in Supporting Information (Fig. S1A), from beginning to 3 hours incubation), and it was subsequently transformed to the oxidized form(AQS_oxi_) after goethite spike (Fig. S1A, from 3 hours to 3.5 hours incubation), indicating the role of AQS in reducing goethite^3^.

In all the reactors spiked with different concentrations of goethite, Fe(II)_tot_ and Fe(II)_dis_ concentrations increased with time and then reached a plateau. The incubation time needed to reach the plateau increased with increasing concentration of the spiked goethite. In reactors containing no more than 0.5 mM goethite, the goethite reduction reached the plateau after about 5 days of goethite reduction (Fig. 1A,B). However, in the reactors containing spiked goethite higher than 2.0 mM, it would take 10 days or more incubation time to reach the plateau (Fig. 1C–E).

In “Strain S12- AQS” pre-incubation reactors, a positive linear relationship exists between the mass-normalized goethite reduction rate and goethite mass-available AQS content (Adj. R-Square = 0.9967) (Fig. S2), which could be due to a change in available surface area per unit goethite mass for redox mediator (AQS) during the subsequent interaction process between redox mediator and iron oxide. AQS content was kept constant at 0.1 mM in each “strain S12- AQS” pre-incubation reactor, while goethite concentration varied from 0.1 to 10.0 mM, which changed the value of goethite mass-available AQS content ([AQS]/[Goethite]). In the reactors containing lower goethite content, more AQS is available for goethite surface site, consequently enhancing the overall potential of electron transfer from the reduced form of AQS to goethite, and increasing the mass-normalized goethite reduction rate. This explained why the reaction time needed for Fe(II) concentration could reach a plateau (dotted line in Fig. 1) increased with increasing content of goethite.

Furthermore, the value of [AQS]/[Goethite] also significantly impacted the relative extent of goethite reduction. Almost 100 percentage of goethite was reduced in the reactor containing 0.1 mM goethite ([AQS]/[Goethite] = 1) while the value in the reactor containing 10.0 mM goethite ([AQS]/[Goethite] = 0.1) reached 32.1 percent at the plateau (Fig. S3, solid symbol).

### Influence of contents of AQS addition to the “strain S12-goethite” pre-incubation reaction systems on microbial goethite reduction

**Figure 2:**
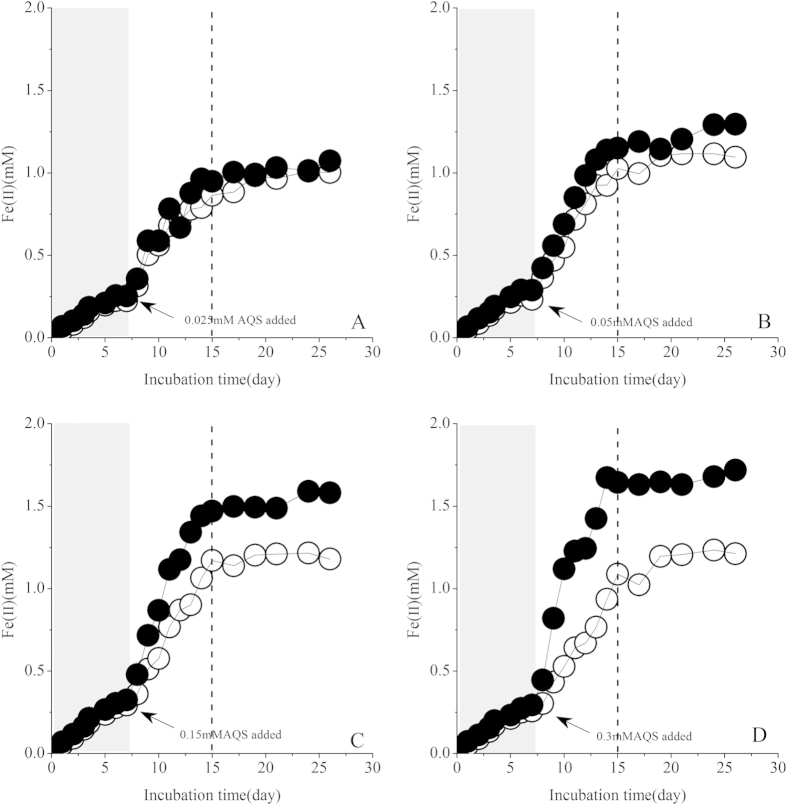
Concentrations of Fe(ΙΙ)_dis_ and Fe(ΙΙ)_tot_ in “strain S12- goethite” pre-incubation reaction systems added with different content of AQS. (Solid symbol: Fe(ΙΙ)_tot_, open symbol: Fe(ΙΙ)_dis_. Reaction systems components: goethite: 2.0 mM; AQS: 0.025 ~ 0.3 mM. The reaction systems containing 2.0 mM goethite inoculated with strain S12 at density of 2.5 × 10^8^ cells·ml^−1^, then after 7 days pre-incubation, different contents (0.025, 0.05, 0.15 and 0.3 mM) of AQS were added to each pre-incubation reaction systems (**A–D**) respectively).

Spiking AQS in the pre-incubation reactors of “strain S12-goethite” resulted in a significant increase in Fe(II)_tot_ and Fe(II)_dis_ concentrations, indicating the increase of goethite reduction rate compared with that before AQS addition (gray area in Fig. 2). The goethite reduction rate increased with the increasing concentration of spiked AQS. For example, the average rate of goethite reduction increased by 3 times after spiking 0.3 mM AQS (Fig. S4). Increasing spiked AQS concentration also increased the extent of goethite reduction, and in the reactor containing a relatively high content of AQS (0.30 mM), nearly 85 percent of goethite was reduced; when the AQS concentration decreased to 0.025 mM, only 53 percentage of goethite were reduced (Fig. S3, open symbol). The rate of goethite reduction decreased sharply after nearly 15th days’ incubation (or 8 days after AQS spike regardless of spiked AQS concentration (dotted lines in Fig. 2A–D), which indicated that the microbial reduction of goethite mediated by AQS reached the equilibrium.

**Figure 3:**
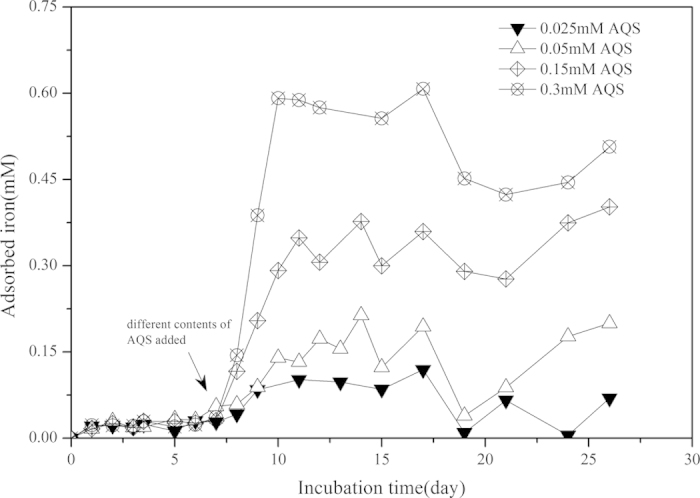
The time course of Fe(ΙΙ)_ads_ contents in “strain S12- goethite” pre-incubation reaction systems added with different concentration of AQS. (Goethite: 2.0 mM; AQS: 0.025 ~ 0.3 mM).

The introduction of redox mediator, AQS, could promote the rate (Fig. S4) and extent of the reductive dissociation of goethite (Fig. S3). Thus the following could be expected to occur: a) Fe(II)_tot_ concentration was increased; b) the enhanced microbially mediated reductive dissolution could result in a smaller size of goethite particle, and subsequently, the specific surface area and adsorption capacity of goethite were enhanced. For example, in “strain S12-goethite” pre-incubation reaction systems containing 0.3 mM AQS, the adsorption capacity of goethite for ferrous iron increased to 1.72 mM·mM^−1^ (Fig. S5), thus, the contents of adsorbed ferrous iron(Fe(II)_ads_) being elevated (Fig. 3). The similar phenomenon could also be seen in “strain S12-AQS” pre-incubation reaction systems (Fig. S6).

### Characteristics of redox mediator transformation in two different pre-incubation reaction systems

The maximum absorbance wavelength of oxidized form of redox mediator (AQS_oxi_) and its hydroquinone reduced form (AQS_red_) were near 329 nm and 380 nm respectively^3^. Considering that the maximum molar absorption coefficients for AQS_oxi_ and AQS_red_ are constants (Table S1), the absorbances at 329 nm and 380 nm will be directly proportional to the concentration of AQS_oxi_ and AQS_red_ respectively.

**Figure 4:**
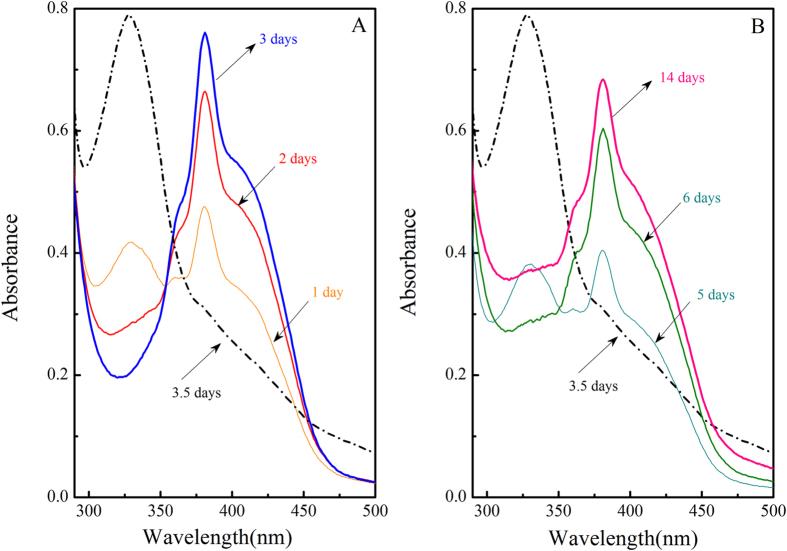
The time courses of the UV/vis spectra of transformation between AQS_red_ and AQS_oxi_ in the typical “strain S12- AQS” pre-incubation reaction system. (The maximum absorbances of AQS_red_ and AQS_oxi_ were at 380 nm and 298 nm respectively, reaction system components: 5.0 mM goethite, 0.1 mM AQS, pH 7.02).

In “strain S12-AQS” pre-incubation reactors, redox mediator was gradually transformed to AQS_red_ within initial 3 days of pre-incubation duration (Fig. 4A). After goethite spike, AQS_red_ decreased sharply and was almost completely transferred to its oxidized form. For example, after 0.5 days incubation with addition of 5.0 mM goethite, the absorbance of 380 nm at which the AQS_red_ has the maximum absorbance was hardly detected (dotted line in Fig. 4). Further investigation of the UV/vis spectra during the subsequent reduction of goethite indicated that the concentration of AQS_red_ increased gradually from 3.5 to 14 days (Fig. 4B). The similar phenomenon could also be seen in other “strain S12-AQS” pre-incubation reaction system containing 0.5 mM goethite (Fig. S1).

**Figure 5:**
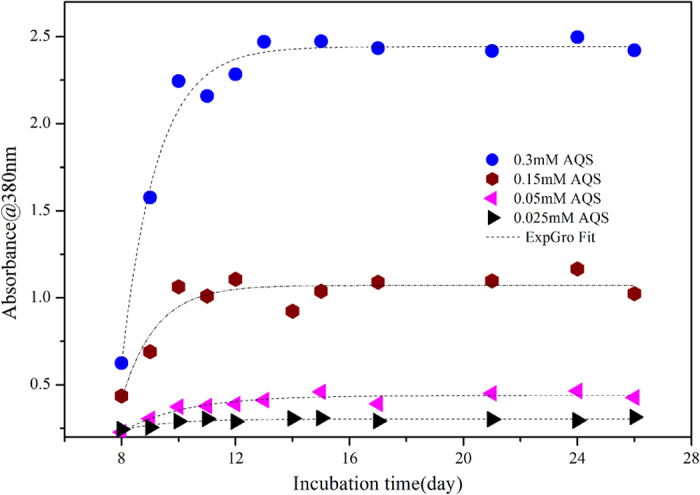
The times course of the maximum absorbance of reductively produced AQS_red_ at 380 nm in “strain S12- goethite” pre-incubation reaction systems added with different concentration of AQS.

In “strain S12-goethite” reactors, after AQS_oxi_ was spiked at different concentrations, the transformation from AQS_oxi_ to AQS_red_ was confirmed by UV-vis absorption spectroscopy (Fig. S7). The temporal change in maximum absorbance of the AQS_red_ at 380 nm could be described by an exponential growth (ExpGro) model(Fig. 5) with the correlation coefficients, Adj. R- Square, were in the range of 0.7768 ~ 0.9762 (Table S2). The fitted ExpGro model was discussed in Text 1 in Supporting Information (Text S1).

## Discussion

**Figure 6:**
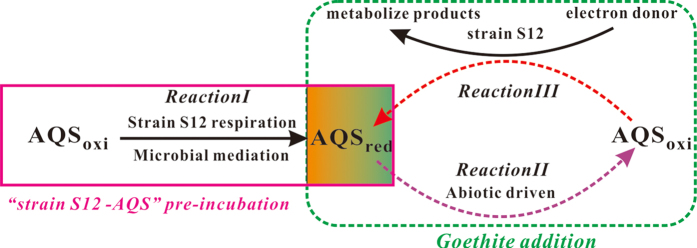
Proposed pathways for the interaction process among AQS, strain S12 and goethite in “strain S12-AQS” pre-incubation reaction systems.

From the analysis of the characteristics of transformation between two redox species of redox mediator in “strain S12-AQS” pre-incubation reaction systems, the transformation process mentioned above could be described by the Fig. 6.

It indicated an obvious reversible redox transformation between two species of redox mediator (Fig. 6), AQS_oxi_ and AQS_red_, with the detailed processes proposed here: 1)microbial mediation pathway(*Reaction І* in Fig. 6): the AQS_oxi_ received the enzymatically produced electrons and was reduced to AQS_red_, which could be accumulated till the equilibrium of the transformation of two redox sensitive species(from beginning to 3 days, Fig. 4A); 2) abiotic pathway(*Reaction ІІ* in Fig. 6): when goethite was introduced to the reaction system, the accumulated AQS_red_ transferred the electrons to goethite surface so as to facilitate the goethite reduction, and meanwhile, AQS_red_ was oxidized to AQS_oxi_ (from 3 days to 3.5 days, Fig. 4A). 3) The produced AQS_oxi_ was subsequently bio-reduced to AQS_red_ during the metabolic process of strain S12(*Reaction ІІІ* in Fig. 6) (from 3.5 days to 14 days, Fig. 4B). Therefore, both biotic and abiotic steps controlled the transformation between two species of the redox mediator, AQS, in the “strain S12- AQS” pre-incubation reaction systems; and consequently, the concentrations of AQS_red_ and AQS_oxi_ varied in different incubation times (Fig. 4).

**Figure 7:**
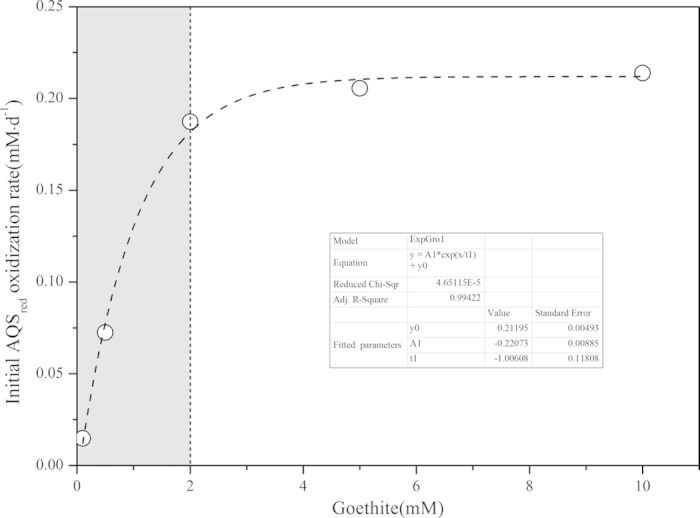
The influence of added goethite contents on the initial AQS_red_ oxidization rate in “strain S12-AQS” pre-incubation reaction systems. (Goethite: 0.1 ~ 10.0 mM; AQS: 0.1 mM; inoculation cell density: 2.5 × 10^8^ cells·ml^−1^).

Further investigation showed that during the transformation process from AQS_red_ to AQS_oxi_ (*Reaction ІІ* in Fig. 6), the relationship between the initial AQS_red_ oxidization rate and total concentration of added goethite could be fitted by the exponential growth model (Adj. R- Square = 0.9942). From the fitted parameters, the initial AQS_red_ oxidization rate reached a maximum at about 0.22 mM·d^−1^ when goethite concentration was high enough to be at 10.0 mM (Fig. 7).

In “strain S12-AQS” pre-incubation reaction systems with the concentration of redox mediator (AQS) kept constant at 0.1 mM, the value of goethite mass-available redox mediator content([AQS]/[Goethite])could be low enough to be at 0.05 or even less in the reaction system containing 2.0 mM goethite or higher. The decreased value of [AQS]/[Goethite] indicated that the redox mediator cannot completely occupy the active surface sites on the goethite surfaces when goethite concentration was increased. Then the influence of goethite concentration on the initial AQS_red_ oxidization rate became not obvious when goethite concentration was higher than 2.0 mM, [AQS]/[Goethite]<0.05, which was in contrast to that in reaction systems containing low concentrations of goethite(range from 0.1 ~ 2.0 mM) (gray area in Fig. 7), and thus the typical saturation-type exponential growth (ExpGro) model could be fitted for the relationship between the initial AQS_red_ oxidization rate and the concentrations of added goethite (Fig. 7).

The similar trend could also be seen in reaction systems with the low inoculation cell density at 5.0 × 10^7^ cells·ml^−1^(Fig. S8), and however, the initial AQS_red_ oxidization rate could only reach a maximum at no more than 0.12 mM·d^−1^ (Fig. S8). Low inoculation cell density was expected to result in decreased microbial activities, and the initial AQS_red_ oxidization rate decreased consequently. The results of fitting Monod type equation of initial microbial goethite reduction rates (Fig. S9) further proved that the degree of biotic reaction increased with increasing inoculation cell density. The fitted maximum initial microbial goethite reduction rate reached 0.5361 mM·d^−1^ (inoculation cell density: 2.5 × 10^8^ cells·ml^−1^) which was nearly twice as much as that with low inoculation cell density(5.0 × 10^7^ cells·ml^−1^)(Table S3).

**Figure 8:**
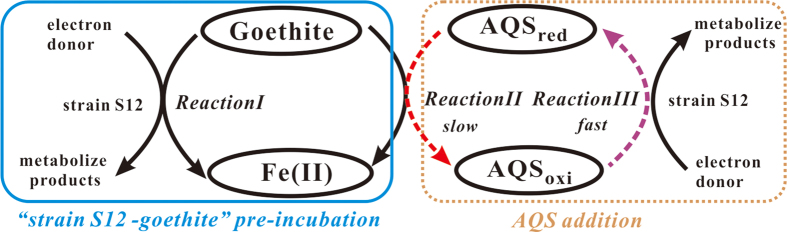
Schematic representations of the proposed interaction process among AQS, strain S12 and goethite in “strain S12-goethite” pre-incubation reaction systems.

When AQS_oxi_ was added to “strain S12-goethite” pre-incubation reaction systems, it received the enzymatically produced electrons and was bio-reduced to AQS_red_, which could subsequently transfer the electrons to goethite surface and enhance the goethite reduction (Fig. 2).The interaction process among redox mediator, strain S12 and goethite could be described by the Fig. 8.

The bio-reduced AQS_red_ was accumulative in the first several incubation days when redox mediator was introduced in “strain S12-goethite” pre-incubation reaction systems (Fig. S7). The result implied that the rate of AQS_red_ formation by the biotic *Reaction ІІІ* in Fig. 8 would be higher than that of AQS_red_ consumption by the abiotic *Reaction ІІ* in Fig. 8, and thus the produced AQS_red_ through *Reaction ІІІ* exceeded that of AQS_red_ oxidized to AQS_oxi_ through *Reaction ІІ*, resulting in the accumulation of bio-reduced AQS_red_ (Fig. 5).

The concentrations of Fe(ΙΙ)_tot_ and Fe(ΙΙ)_dis_ in “strain S12-goethite” pre-incubation reaction systems increased when different concentrations of redox mediator were introduced, and eventually they leveled off after about 15 days incubation period (Fig. 2), based on Fig. 8, it indicated that the rate of microbial goethite reduction (*Reaction І*) and redox mediator-assistant abiotic goethite reduction (*Reaction ІІ*) both decreased and almost got equilibrium. During the course of rate decreasing of *Reaction ІІ* in Fig. 8, the remaining AQS_oxi_ in reaction systems would be transformed to AQS_red_ driven by *Reaction ІІІ* in Fig. 8, and subsequently the content of AQS_red_ would reach a plateau (Fig. 5) indicating that the transformation between AQS_oxi_ and AQS_red_ under biotic driven was in equilibrium.

A spontaneous reaction occurs in the direction of increasing the reduction potential (ΔE). The reduction potentials of *Reaction ІІ* in Fig. 8, could be calculated using the Nernst equation, allow the energetics of each half-reaction to be evaluated separately (details seen in Text S2):


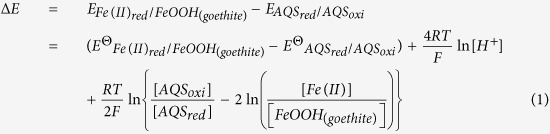


where 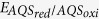 and 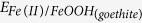 correspond to the apparent reduction potential of each redox couple at given conditions. 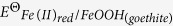
and 
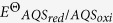
, the standard reduction potentials at given pH are constant, then, based on Eq. 1, the ΔE value was determined by the values of 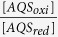 and 
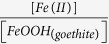
.

**Figure 9:**
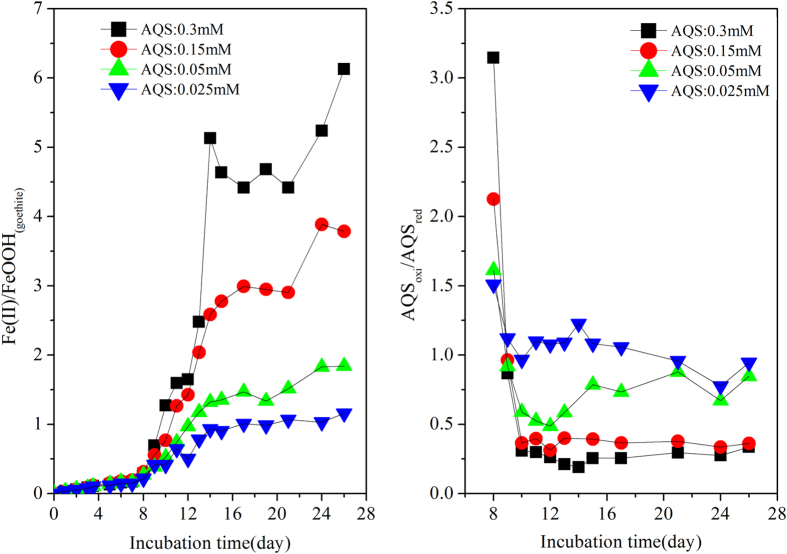
The incubation times course of the values of 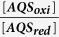 and 
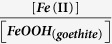
 in “strain S12- goethite” pre-incubation reaction systems.

With increasing content of AQS added to the “strain S12-goethite” pre-incubation reaction systems, the value of 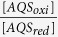 decreased, while the value of 
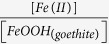
 increased (Fig. 9). Based on Eq. 1, the value of _Δ*E*_ decreased with the increasing contents of AQS added to reaction system. Since a decreasing reduction potential means that reduction reaction approaches equilibrium and it occurs more difficultly, that is to say, the reduction reaction rate of *Reaction ІІ* in Fig. 8 decreases consequently with the increasing contents of AQS added to reaction system.

**Figure 10:**
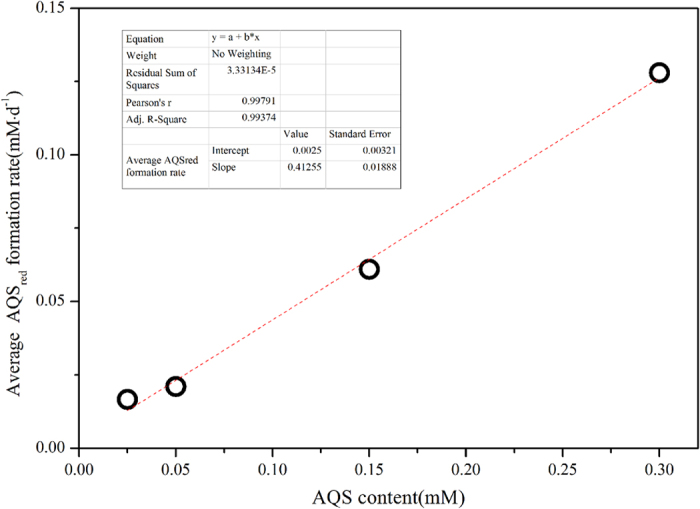
The influence of added redox mediator contents on the initial AQS_red_ formation rate in “strain S12-goethite” pre-incubation reaction systems.

During the course of the reduction reaction rate decreasing of *Reaction ІІ* in Fig. 8, that is to say, the rate of the AQS_red_ consumed by *Reaction ІІ* decreased. However, the AQS_red_ was continually produced from *Reaction ІІІ* in Fig. 8, it will accumulate and subsequently the concentration of AQS_red_ would reach a plateau. In each “strain S12-goethite” pre-incubation reaction systems, the inoculation cell density was kept constant and microbial activities could be assumed equal. Thus the concentration of AQS would be the rate limiting factor for biotic *Reaction ІІІ* in Fig. 8 which driven the transformation from AQS_oxi_ to AQS_red_. Then the average apparent AQS_red_ formation rate through *Reaction (ІІІ)* in Fig. 8 would be expected to increase with increasing concentration of AQS (Fig. 10), and a positive linear relationship with the correlation coefficients of 0.9937 was found between AQS contents and the average AQS_red_ formation rate in “strain S12-goethie” pre-incubation reaction systems.

Organic matters are redox active natural macromolecules that are ubiquitous in soils and sediments and predominate in the subsurface. The redox activity of organic matter has been primarily ascribed to quinone-hydroquinone moieties. In environments with variable redox conditions, organic matters containing a pool of functional moieties have reversible electron transfer ability and can act as redox mediator^26^. Thus organic matters shuttle electrons from microbe to poorly accessible mineral phases, such as iron oxides, could not only act as redox buffers by accepting electrons from microbial respiration under anoxic condition but also mediate biogeochemical redox reactions^27^. These results of our current investigation will help to understand the microbial reduction of the Fe(III) oxide, especially in natural environment containing various organic matters, which can act as the potential electron acceptor or donor to facilitate the interaction between microbe and Fe(III) oxides.

## Methods

The preparation of deoxygenated distilled deionized water, properties of chemicals electrolyte solutions, and anaerobic glove box were provided in Text S3.

### Goethite synthesis and SEM, XRD analysis

Goethite was used in the experiments as the model Fe(III) oxide since it is commonly found in environment. Goethite was synthesized using a method described by Schwertmann and Cornell^28^. Details of the syntheses, washing, and characterization of the goethite were provided in the Text S4 and Figs S10 and S11. Briefly, 120 ml of 1 M Fe(NO_3_)_3_ was mixed with 200 ml of 4.5 M KOH solution, and the mixture was then boiled at 70 °C for 60 hs by constant stirring. After being cooled down to the room temperature, the mixture was filtered, and the yellowish precipitates on the filter were washed repeatedly using deionized water to remove any dissolved salts. The goethite slurries were diluted to 200.0 mM in water and were sonicated for 1 h to disperse the lyophilized particles.

### Redox mediator and its UV-vis spectra

Anthraquinone-2-sulfonate(AQS) was used as the model redox mediator(Table S1) due to its high water solubility, and the convenience of analysis using UV-visible spectrophotometry. AQS has oxidized and reduced forms as quinone and hydroquinone moieties, respectively. The maximum absorbances of the oxidized form (AQS_oxi_) and reduced form ( AQS_red_) were at 329 and 380 nm wavelength, respectively. The redox transformation between AQS_red_ and AQS_oxi_ can be determined using UV-vis absorption spectroscopy. Procedures for obtaining absorption spectra of different forms of redox mediator (AQS_red_ and AQS_oxi_) were reported in our previous study^3^. Briefly, 1.5 ml suspension in a reactor was transferred to 2 ml centrifuge tube, and centrifuged for 2 minutes at 13000 rpm. The supernatant containing redox mediator was collected by a quartz cuvette and was quickly scanned using a UV-visible spectrophotometer (DR 5000, HACH™, USA).

### Bacteria and medium

*Shewanella decolorationis* S12 (strain S12), a new species of the genus *Shewanella*, was provided courtesy by Dr. Xu (Guangdong Provincial Key Laboratory of Microbial Culture Collection and Application, Guangzhou, China). Strain S12 was isolated from the activated sludge of a textile printing wastewater treatment plant in Guangzhou, China. Strain S12 can grow in the presence of various electron acceptors, such as oxygen, nitrate, nitrite, ferric iron and sulfite, showing remarkable respiratory versatility^29^, as do other members of DIRB^30^,^31^.

Strain S12 was routinely cultured aerobically at 32 °C in Luria-Bertani (LB) medium. The LB medium contains tryptone(10.0 g·L^−1^), yeast extract(5.0 g· L^−1^),NaCl(10.0 g·L^−1^) at pH 7.0 (adjusted with 5 M NaOH), and was sterilized by autoclaving for 30 mins. The strain was harvested from the LB cultures at mid to late log phase by centrifuging the suspension for 10 mins at 5 °C. The cells were washed with a pH buffer (1,4- piperazinediethanesulfonic acid (PIPES) buffer, 15.0 mM, pH 7.02) to remove residual medium, and resuspended in the buffer.

### Experiment design and procedures

In natural environment, it is expected that indigenous bacteria and Fe(III) oxide minerals(electron acceptors) or redox sensitive humic substance(redox mediator) are co-present for an extended period of time prior to the interaction among “redox mediator-iron oxide-DIRB”. Based on these considerations, the following sets of experiments were designed and performed:

1. “strain S12- redox mediator” was first incubated in solutions containing strain S12 and 0.1 mM AQS for 3 days, and this incubated system was then spiked with goethite at variable concentrations (0.1 ~ 10.0 mM) to examine the possible transformation pathway of AQS during the interaction process of AQS-goethite.
2. “strain S12-goethite” was first incubated in solutions containing strain S12 and 2.0 mM goethite for 7 days, and the incubated system was then spiked with AQS_oxi_ at variable concentrations (0.025 ~ 0.30 mM) to evaluate subsequent AQS-goethite interactions.

In a typical experiment, various volumes of goethite slurry and lactate were mixed to achieve desired concentrations, followed by addition of PIPES buffer solution (15.0 mM, pH 7.02), AQS (0.1 mM) and NH_4_Cl (20.0 mM). Tubes were purged with N_2_ and sealed with thick butyl rubber stoppers. Strain S12 cell suspension (D600 nm = 1.75) was added from a freshly washed culture in PIPES buffer system using a needle and syringe purged with N_2_ to obtain a final cell concentration at 2.5 × 10^8^ cells·ml^−1^. Low inoculation cell density at 5.0 × 10^7^ cells·ml^−1^ was also used in parallel experiments to evaluate the effect of cell density on the AQS-Goethite interactions. If not specifically mentioned, inoculation cell density was 2.5 × 10^8^ cells·ml^−1^ with all bacterial experiments being incubated at 32 °C.

### Definition of different species of reductively produced ferrous iron

The suspension samples were taken at selected times, being filtered to measure dissolved Fe(II) using the ferrozine method^32^. The total Fe(II) was measured by acid-extraction (0.5 M HCl) for one hour. The extracted Fe(II) was then measured by the ferrozine method. The difference between total and dissolved Fe(II) is the sorbed Fe(II) in the suspensions.

For the convenience of discussion, different species of reductively produced ferrous iron were defined as Eq. 2:


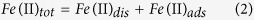


where Fe(ΙΙ)_dis_ is dissolved Fe(II) as measured in filtered (0.45 μm) samples, Fe(ΙΙ)_tot_ is the total Fe(II) extracted by 0.5 M HCl, and Fe(ΙΙ)_ads_ is the adsorbed ferrous iron calculated from the difference of the Fe(ΙΙ)_dis_ and Fe(ΙΙ)_tot_. All the procedures were performed in an anoxic chamber (Bactron ΙΙΙ, SHELLAB™, USA).

## Additional Information

**How to cite this article**: Zhu, W. *et al*. Characteristics and Kinetic Analysis of AQS Transformation and Microbial Goethite Reduction:Insight into “Redox mediator-Microbe-Iron oxide” Interaction Process. *Sci. Rep.* **6**, 23718; doi: 10.1038/srep23718 (2016).

## Supplementary Material

Supplementary Information

## References

[b1] Weber K. A., Achenbach L. A. & Coates J. D. Microorganisms pumping iron: anaerobic microbial iron oxidation and reduction. Nat. Rev. Microbiol. 4, 752–764 (2006). 16980937 10.1038/nrmicro1490

[b2] Richter K., Schicklberger M. & Gescher J. Dissimilatory reduction of extracellular electron acceptors in anaerobic respiration. Appl. Environ. Microbiol. 78, 913–921 (2012). 22179232 10.1128/AEM.06803-11 PMC3273014

[b3] Zhu W., Nan Y., Huang T. & Wu F. The mechanism, thermodynamic and kinetic characteristics of the microbial reduction of goethite mediated by anthraquinone-2-sulfonate. Geomicrobiol. J. 30, 928–940(2013).

[b4] Piepenbrock A., Schröder C. & Kappler A. Electron transfer from humic substances to biogenic and abiogenic Fe(ΙΙΙ) oxyhydroxide minerals. Environ. Sci. Technol. 48, 1656–1664 (2014). 24400782 10.1021/es404497h

[b5] Royer R. A. . Enhancement of hematite bioreduction by natural organic matter. Environ. Sci. Technol. 36, 2897–2904 (2002). 12144265 10.1021/es015735y

[b6] Roden E. E. In Kinetics of water-rock interaction (eds Brantley S. L., Kubicki J. D. & White A. F.) Ch. 8, 335–415 (Springer, New York, 2008).

[b7] Amstaetter K., Borch T. & Kappler A. Influence of humic acid imposed changes of ferrihydrite aggregation on microbial Fe(III) reduction. Geochim. Cosmochim. Acta 85, 326–341 (2012).

[b8] Jiang J. & Kappler A. Kinetics of microbial and chemical reduction of humic substances: implications for electron shuttling. Environ. Sci. Technol. 42, 3563–3569 (2008). 18546690 10.1021/es7023803

[b9] O’Loughlin E. J. Effects of electron transfer mediators on the bioreduction of lepidocrocite (γ-FeOOH) by *Shewanella putrefaciens* CN32. Environ. Sci. Technol. 42, 6876–6882 (2008). 18853803 10.1021/es800686d

[b10] Li X., Liu T., Liu L. & Li F. Dependence of the electron transfer capacity on the kinetics of quinone-mediated Fe(ΙΙΙ) reduction by two iron/humic reducing bacteria. RSC Adv. 4, 2284–2290 (2014).

[b11] Watanabe K., Manefield M., Lee M. & Kouzuma A. Electron shuttles in biotechnology. Curr. Opin. Biotechnol. 20, 633–641 (2009). 19833503 10.1016/j.copbio.2009.09.006

[b12] Van der Zee F. P. & Cervantes F. J. Impact and application of electron shuttles on the redox (bio)transformation of contaminants: a review. Biotechnol. Adv. 27, 256–277 (2009). 19500549 10.1016/j.biotechadv.2009.01.004

[b13] Uchimiya M. & Stone A. T. Reversible redox chemistry of quinones: impact on biogeochemical cycles. Chemosphere 77, 451–458 (2009). 19665164 10.1016/j.chemosphere.2009.07.025

[b14] Skancke A. & Skancke P. N. In The quinonoid compounds (Eds. Patai S. & Zvi Rappoport Z.) Vol. 1, 1–28 (John Wiley & Sons, 1988).

[b15] Shi Z. . Redox reactions of reduced flavin mononucleotide (FMN), riboflavin (RBF), and anthraquinone-2,6-disulfonate (AQDS) with ferrihydrite and lepidocrocite. Environ. Sci. Technol. 46, 11644–11652(2012). 22985396 10.1021/es301544b

[b16] Zachara J. M. . The mineralogic transformation of ferrihydrite induced by heterogeneous reaction with bioreduced anthraquinone disulfonate (AQDS) and the role of phosphate. Geochim. Cosmochim. Acta 75, 6330–6349 (2011).

[b17] Shi Z., Zachara J. M., Wang Z., Shi L. & Fredrickson J. K. Reductive dissolution of goethite and hematite by reduced flavins. Geochim. Cosmochim. Acta 121, 139–154 (2013).

[b18] Liu C., Zachara J. M., Foster N. S. & Strickland J. Kinetics of reductive dissolution of hematite by bioreduced anthraquinone-2,6-disulfonate. Environ. Sci. Technol. 41, 7730–7735 (2007). 18075081 10.1021/es070768k

[b19] Orsetti S., Laskov C. & Haderlein S. B. Electron transfer between iron minerals and quinones: estimating the reduction potential of the Fe(II)-goethite surface from AQDS speciation. Environ. Sci. Technol. 47, 14161–14168 (2013). 24266388 10.1021/es403658g

[b20] Uchimiya M. & Stone A. T. Redox reactions between iron and quinones: thermodynamic constraints. Geochim. Cosmochim. Acta 70, 1388–1401 (2006).

[b21] Ruebush S. S., Icopini G. A., Brantley S. L. & Tien M. *In vitro* enzymatic reduction kinetics of mineral oxides by membrane fractions from *Shewanella oneidensis* MR-1. Geochim. Cosmochim. Acta 70, 56–70 (2006).

[b22] Zegeye A., Ruby C. & Jorand F. Kinetic and thermodynamic analysis during dissimilatory γ-FeOOH reduction: formation of green rust 1 and magnetite. Geomicrobiol. J. 24, 51–64 (2007).

[b23] Bonneville S., Behrends T., Cappellen P. V., Hyacinthe C. & Röling W. F. M. Reduction of Fe(III) colloids by *Shewanella putrefaciens*: a kinetic model. Geochim. Cosmochim. Acta 70, 5842–5854 (2006).

[b24] Burgos W. D. . Reaction-based modeling of quinone-mediated bacterial iron(III) reduction. Geochim. Cosmochim. Acta 67, 2735–2748, (2003).

[b25] Melton E. D., Swanner E. D., Behrens S., Schmidt C. & Kappler A. The interplay of microbially mediated and abiotic reactions in the biogeochemical Fe cycle. Nat. Rev. Micro. 12, 797–808 (2014). 10.1038/nrmicro3347 25329406

[b26] Lovley D. R., Fraga J. L., Coates J. D. & Blunt-Harris E. L. Humics as an electron donor for anaerobic respiration. Environ. Microbiol. 1, 89–98 (1999). 11207721 10.1046/j.1462-2920.1999.00009.x

[b27] Roden E. E. . Extracellular electron transfer through microbial reduction of solid-phase humic substances. Nature Geosci. 3, 417–421 (2010).

[b28] Schwertmann U. & Cornell R. M. In Iron Oxides in the laboratory: preparation and characterization, Edn. 2^nd^. Ch. 5, 67–92 (Wiley-VCH Verlag GmbH, 2000).

[b29] Hong Y. . Respiration and growth of *Shewanella decolorationis* S12 with an azo compound as the sole electron acceptor. Appl. Environ. Microbiol. 73, 64–72 (2007). 17085710 10.1128/AEM.01415-06 PMC1797134

[b30] Klonowska A., Heulin T. & Vermeglio A. Selenite and tellurite reduction by *Shewanella oneidensis*. Appl. Environ. Microbiol. 71, 5607–5609 (2005). 16151159 10.1128/AEM.71.9.5607-5609.2005 PMC1214622

[b31] Hau H. H. & Gralnick J. A. Ecology and biotechnology of the genus *Shewanella*. Annu. Rev. Microbiol. 61, 237–258 (2007). 18035608 10.1146/annurev.micro.61.080706.093257

[b32] Stookey L. L. Ferrozine—a new spectrophotometric reagent for iron. Anal. Chem. 42, 779–781 (1970).

