# Supplementary material for: Characteristics and Kinetic Analysis of AQS Transformation and Microbial Goethite Reduction:Insight into “Redox mediator-Microbe-Iron oxide” Interaction Process
Source: Sci Rep. 2016 Mar 29;6:23718. doi: 10.1038/srep23718 (PMC4810424; doi:10.1038/srep23718)
Supplement: Supplementary Information [file srep23718-s1.pdf]

Supporting Materials

Characteristics and Kinetic Analysis of AQS Transformation and Microbial Goethite Reduction:Insight into "Redox mediator-Microbe-Iron oxide" Interaction Process

Weihuang Zhu[*1], Mengran Shi[1], Dan Yu[1], Chongxuan Liu[2],Tinglin Huang[1], Fengchang Wu[3]

[1]Key Laboratory of Northwest Water Resources, Environment and Ecology, Ministry of Education, Xi'an University of Architecture and Technology, Xi'an 710055, China

[2]Pacific Northwest National Laboratory, Richland, Washington 99352, United States

[3]State Key Laboratory of Environmental Criteria and Risk Assessment, Chinese Research Academy of Environmental Sciences, Beijing 100012, China

∗ Corresponding author: email: zhuweihuang@gmail.com, Phone: (86)29-82202729, fax: (86)29-82202729.

21 pages

---

4 Texts

3 Tables

11 Figures

Text S1. The fitted exponential growth (ExpGro) model for the time course of absorbance@380nm of AQS $_{red}$

After addition of redox mediator, AQS_{red} could be subsequently bio-reductively produced during the strain S12 metabolic process of (reaction III in Fig.8 in manuscript), it could be denoted by the following *equations:*

$$AQS_{oxi} + strain\ S12 \xrightarrow[reaction\ III]{+e} AQS_{red} + metabolized\ products \quad (1)$$

$$AQS_{red} + goethite \xrightarrow[reaction\ II]{+e} AQS_{oxi} + Fe(\mathrm{II})_{dis} + Fe(\mathrm{II})_{ads} \quad (2)$$

and the initial AQS_{red} formation rate could be written as:

$$\frac{d[AQS_{red}]}{d[t]} = k_{\mathrm{III}}[AQS_{oxi}][strain\ S12] - k_{\mathrm{II}}[AQS_{red}][goethite] \quad (3)$$

Where t, k_{II} and k_{III} denoted the reaction time and rate constants for reaction II and reaction III respectively, since the content of goethite and inoculation cell density were kept constant at 2.0 mM and 2.5×10^{8} cells·ml^{-1} respectively in "strain S12-goethie" pre-incubation reaction system, then *equation* 3 could be written as:

$$\begin{aligned} \frac{d[AQS_{red}]}{d[t]} &= k_{\mathrm{III}}[C_0 - AQS_{red}][strain\ S12] - k_{\mathrm{II}}[AQS_{red}][goethite] \\ &= k_{\mathrm{III},0}[AQS_{red}] + k_{\mathrm{II},0}[AQS_{red}] \\ &= (k_{\mathrm{III},0} + k_{\mathrm{II},0})[AQS_{red}] \\ &= k_0[AQS_{red}] \end{aligned} \quad (4)$$

Where C_0 denoted the initial contents of redox mediator added to the reaction systems, $k_{\mathrm{II},0}$, $k_{\mathrm{III},0}$ and k_0 denoted the pseudo first order rate constants for reaction II, reaction III and overall reaction rate constant for AQS_{red} formation respectively.

From the *equation* 4:

$$\frac{d[AQS_{red}]}{[AQS_{red}]} = k_0 d[t] \quad (5)$$

$$\ln(AQS_{red}) = k_0 t + c \quad (6)$$

Where c was a constant related to the corresponding reaction system, and *equation* 6

could be integrated as:

$$AQS_{red} = A \cdot \exp(x / t_1) + m_1 \quad (7)$$

where x denoted the incubation time(day) , the absolute value of constants for A and t_1 could reflect the inherent qualities of different reaction systems, and the value of constants, m_1, denoted the content of AQS_{red} at final equilibrium state.

According to Lambert-Beer law:

$$Abs_{@380nm} = \varepsilon \cdot l \cdot [AQS_{red}] \quad (8)$$

Where $Abs_{@380nm}$ is the maximum absorbance of the AQS_{red} at 380 nm. l is the light path length, ε is the absorption coefficients. From *Equation* 7 and 8:

$$Abs_{@380nm} = \varepsilon \cdot l \cdot A \cdot \exp(x / t_1) + \varepsilon \cdot l \cdot m_1 = A_0 \cdot \exp(x / t_1) + m_0 \quad (9)$$

where m_0, denoted the absorbance@380nm of AQS red at final equilibrium state. *Equation 9* indicated the incubation time(t_1) course of absorbance@380nm of AQS_{red} was typical of ExpGro model, which was further proved by the experimental data(Fig.5 in manuscript).

Text S2. The reduction potentials involved in interaction process of "goethite-redox mediator" in"strain S12-goethite" pre-incubation reaction systems

When AQS was added to "strain S12-goethite" pre-incubation reaction systems, it received the electronic enzymatically produced and bio-reduced to AQS_{red}, which could transfer the electronics to goethite surface and enhance the goethite reduction (Fig.2 in manuscript). The *Reaction* II in Fig.8 (in manuscript) could be expressed:

$$AQS_{red} + 2FeOOH_{(goethite)} + 4H^{+} \rightarrow AQS_{oxi} + 2Fe(\mathrm{II}) + 4H_2O \quad (10)$$

The redox *Equation* 10 could be separated into two half reactions for quinone (Eq. 11) and iron (Eq. 12):

Reduction half-reaction: $FeOOH_{(goethite)} + 3H^{+} + e \rightarrow Fe(\mathrm{II}) + 2H_2O \quad (11)$

Oxidation half-reaction: $AQS_{red} \rightarrow AQS_{oxi} + 2e^{-} + 2H^{+}$ (12)

The reduction half-reaction pair with higher reduction potential is thermodynamically capable of oxidizing the oxidation half-reaction pair with the lower reduction potential. The Nernst equation for each of these reduction potential of half-reaction can be written as follows:

$$E_{Fe(\mathrm{II})/FeOOH_{(goethite)}} = E^{\ominus}{}_{Fe(\mathrm{II})/FeOOH_{(goethite)}} - \frac{RT}{F}\ln\left(\frac{[Fe(\mathrm{II})]}{[FeOOH_{(goethite)}][H^{+}]^{3}}\right) \quad (13)$$

$$E_{AQS_{red}/AQS_{oxi}} = E^{\ominus}{}_{AQS_{red}/AQS_{oxi}} - \frac{RT}{2F}\ln\left(\frac{[AQS_{oxi}][H^{+}]^{2}}{[AQS_{red}]}\right) \quad (14)$$

where $E^{\ominus}{}_{Fe(\mathrm{II})/FeOOH_{(goethite)}}$ and $E^{\ominus}{}_{AQS_{red}/AQS_{oxi}}$ are the standard reduction potentials at given pH; Fe(II) and $FeOOH_{(goethite)}$ represent the activities of ferrous and ferric ions associated with the goethite.

ΔE, the reduction potential, could be calculated using the Nernst equation, allow the energetics of each half-reaction to be evaluated separately:

$$\Delta E = E_{Fe(\mathrm{II})/FeOOH_{(goethite)}} - E_{AQS_{red}/AQS_{oxi}} \quad (15)$$

Where $E_{AQS_{red}/AQS_{oxi}}$ and $E_{Fe(\mathrm{II})/FeOOH_{(goethite)}}$ correspond to the apparent reduction potential of each redox couple at given conditions. A spontaneous reaction occurs in the direction of increasing the apparent reduction potential, when the reaction is at equilibrium, the ΔE is zero. Based on *equation* 13 ~ 15, equation 16 could be acquired:

$$\Delta E = (E^{\ominus}{}_{Fe(\mathrm{II})_{red}/FeOOH_{(goethite)}} - E^{\ominus}{}_{AQS_{red}/AQS_{oxi}}) + \frac{4RT}{F}\ln[H^{+}] + \frac{RT}{2F}\ln\left\{\frac{[AQS_{oxi}]}{[AQS_{red}]} - 2\ln\left(\frac{[Fe(\mathrm{II})]}{[FeOOH_{(goethite)}]}\right)\right\} \quad (16)$$

where $E^{\ominus}_{Fe(\mathrm{II})_{red}/FeOOH_{(goethite)}}$ and $E^{\ominus}_{AQS_{red}/AQS_{oxi}}$ were the standard reduction potential at given pH(set at 7.02 by PIPES buffer).

Text S3. Water, bottles, anaerobic chamber, chemicals and reagents

All solutions were prepared from reagent grade chemicals and were used as received, unless otherwise stated. Distilled, deionized water (DDW) with a resistivity of 18 MΩ-cm (Barnstead nanopure) were used for all experiments. Bottles and glassware were soaked in 2.0 M hydrochloric acid (Sigma-Aldrich), rinsed with distilled water, soaked in 5.0 M nitric acid (Fisher Scientific), rinsed with distilled water, and then rinsed with DDW water and air dried.

Experiments were mainly conducted inside an anaerobic chamber (Bactron III, SHELLAB™, USA). Deoxygenated, distilled, deionized water (DDDW) was prepared by boiling the DDW in a 2.0 L bottle under the vacuum for 2.0 hours, then transferring the water inside the anaerobic chamber, and sparging with high purity nitrogen gas for overnight.

Text S4. Goethite synthesis and characterization

Goethite synthesis

Goethite suspended particles were synthesized according to methods of Schwertmann and Cornell[1]. Briefly, 180 mL of 5.0 M KOH solution were added rapidly with stirring to 100 mL of 1 M $Fe(NO_3)_3$ solution in a 2.0 L polyethylene flask. The resulting suspension was then brought to 2.0 L total volume with purity water and heated at 70 ℃ for 60 h. The precipitated product was centrifuged, washed with high-purity water 10 times repeatedly to remove salts. The synthesized goethite was confirmed using powder X-ray diffraction and scan electron microscopy (Figure S1 and Figure S2).

SEM and XRD analysis

Scanning Electron Microscope (JSM-6510LV, JEOL™, JAPAN) was used to analyze the cell-goethite spatial relationship at an accelerating voltage of 15-20 keV. First, the mineral-cell suspension was fixed in 2.5% glutaraldehyde for at least 24

hours, then was subjected to dehydration using elevated concentrations of ethanol, followed by critical point drying and coating with a 20 nm gold layer, and then was ready for SEM observation.

Goethite suspension was filtered through a 0.45μm membrane in the anoxic chamber; the wet paste was spread out on a glass plate and coated with glycerol to prevent oxidation. The XRD data were collected with a D/MAX-2400 diffractometer. The X-ray source was a Cu anode (λ=0.15405 nm). The diffractgram was recorded in the 3~90° 2θ range with a 0.02° step size.

Table S1. Chemical Structures and relevant physicochemical properties of redox mediator (anthraquinone- 2-sulfonate, AQS) and its redox transformation reaction

Two-electron half reaction	Reduction potential (mv)		pK_a		$\varepsilon(M^{-1}cm^{-1})$ [d]	
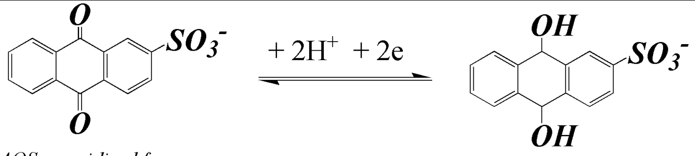 $+2H^+ + 2e$	E^{0} [a]	$E^{0\prime}$ [b]	pK_{a1} [c]	pK_{a2} [c]	AQS_{oxi}	AQS_{red}
AQS_{oxi} ,oxidized form maximum absorbance wavelength：329n*m*; AQS_{red} ,reduced form maximum absorbance wavelength：380n*m*	-460	-600	7.68	10.92	5054 @329nm	5799 @380nm

[a] Half-wave potential (from CV), vs SCE, value was calculated using the Nernst equation(pH 7.0, 25 ℃.),reference [2, 3]
[b] $E^{\circ\prime}$ is the formal potential
[c] *K*a1, *K*a2 are the nonthermodynamic (concentration-based) acid dissociation constants[2]
[d] Extinction coefficients at the maximum absorption wavelength measured in the present work

Table S2 Fitted parameters of ExpGro kinetic mode for relationship between AQS_{red} content (mM) and incubation time(day)

Redox mediator content/mM	A	t_1	m_1	Adj. R-Square
0.025	-7.191±12.74	-1.689±0.618	0.3038±0.045	0.7768
0.05	-7.606±6.910	-2.225±0.546	0.439±0.011	0.8864
0.15	-539.2±1142.0	-1.192±0.371	1.071±0.034	0.8395
0.3	-1180.8±873.7	-1.235±0.139	2.443±0.035	0.9762

Table S3 Fitted parameters of the Monod kinetic mode

Inoculation density: 2.5×10^8 cells·ml^{-1}			Inoculation density: 5×10^7 cells·ml^{-1}		
V_{max} (mM·day^{-1})	K_m (mM)	Adj. R-Square	V_{max} (mM·day^{-1})	K_m (mM)	Adj. R-Square
0.5361±0.033	1.745±0.3561	0.9864	0.2529±0.015	0.8526±0.1307	0.9933

Figure S1 The typical UV/vis spectra of oxidized or reduced forms of AQS during the transformation process

(The spectroscopies were acquired in “strain S12- AQS” pre-incubation reaction systems with AQS content at 0.1mM. After 3 days incubation, 0.5mM goethite was added to reaction systems. the maximum absorbances of AQS_{red} and AQS_{oxi} are at 380 nm and 298 nm respectively)

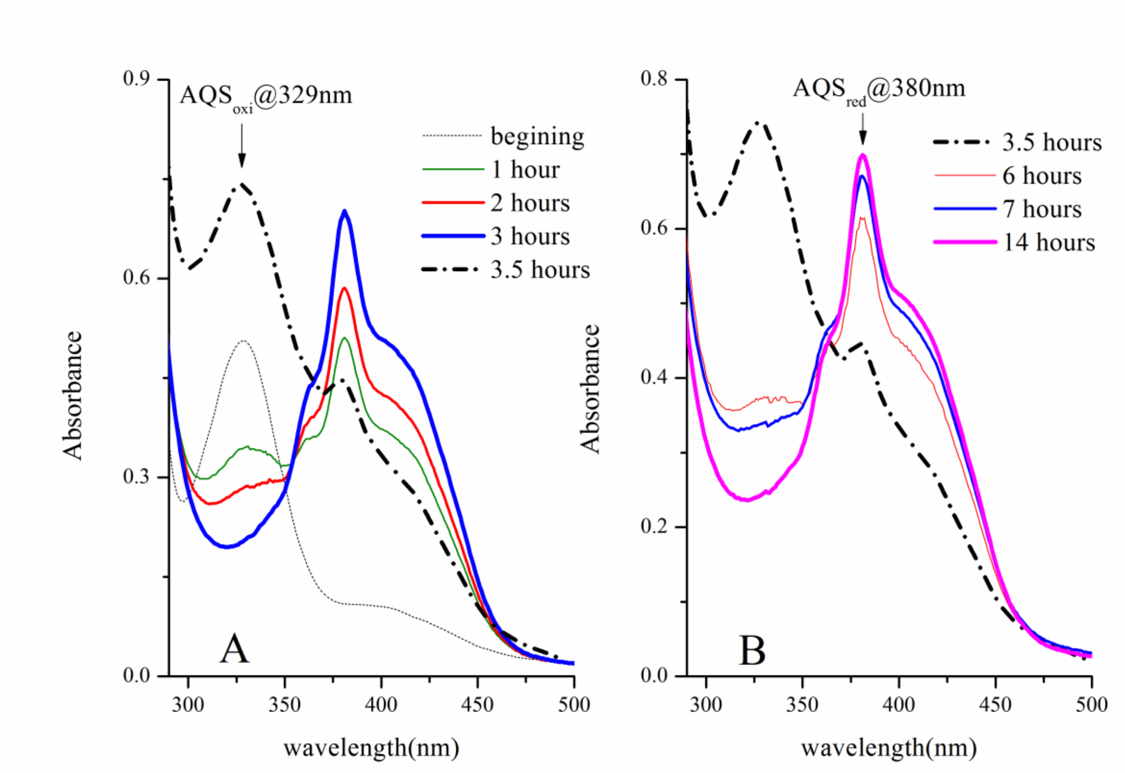

Figure S2 Relationship between goethite mass-normalized reduction rate and goethite mass-available AQS content in "strain S12- AQS" pre-incubation reaction systems

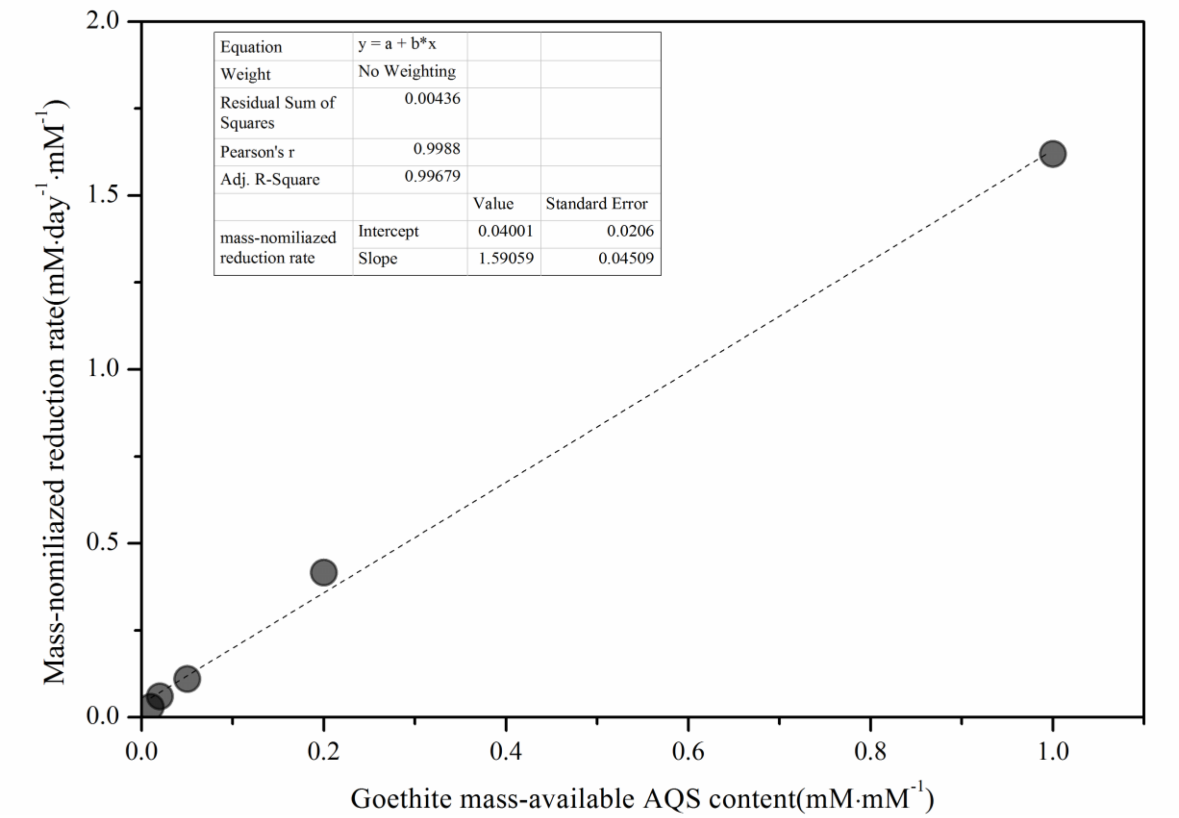

Figure S3 The influence of contents of goethite and AQS on the extent of microbial goethite reduction in “strain S12- goethite” and “strain S12- AQS” pre-incubation reaction systems respectively

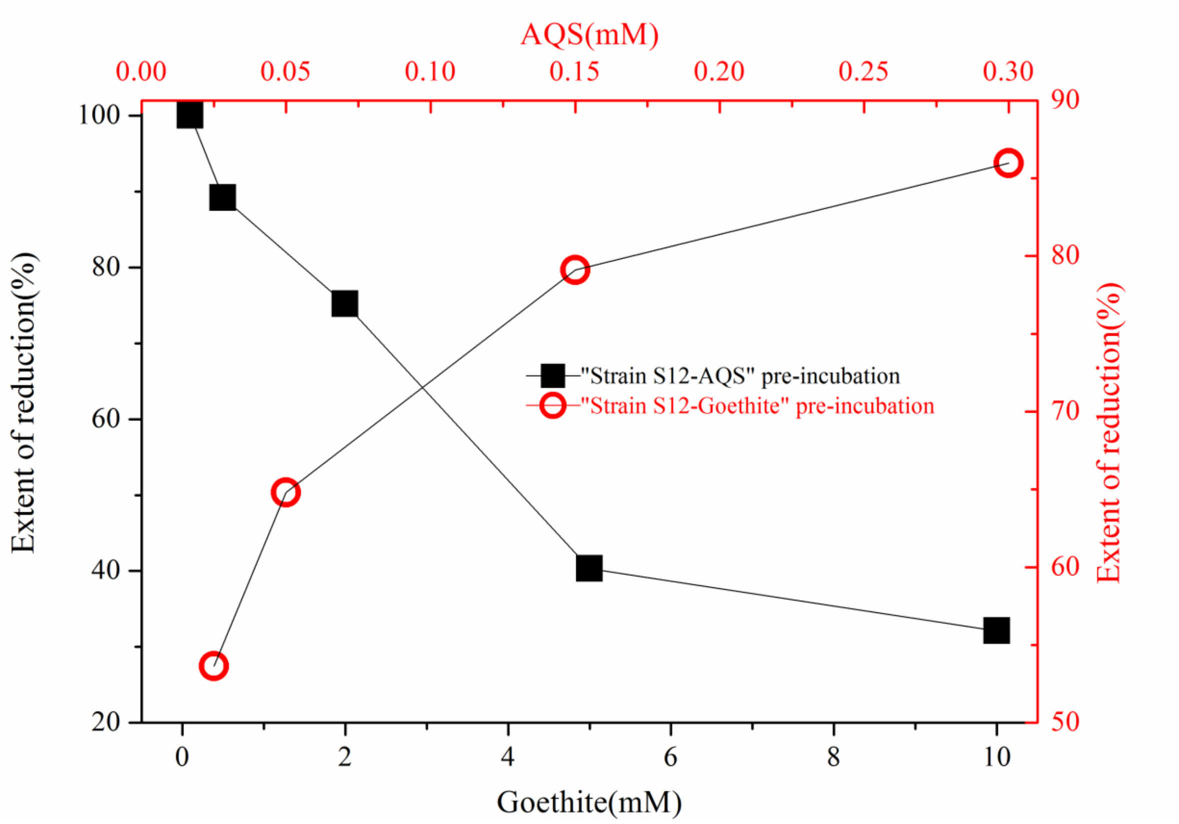

Figure S4 The influence of added AQS contents on the microbial goethite reduction rate in "strain S12- goethite" pre-incubation reaction systems

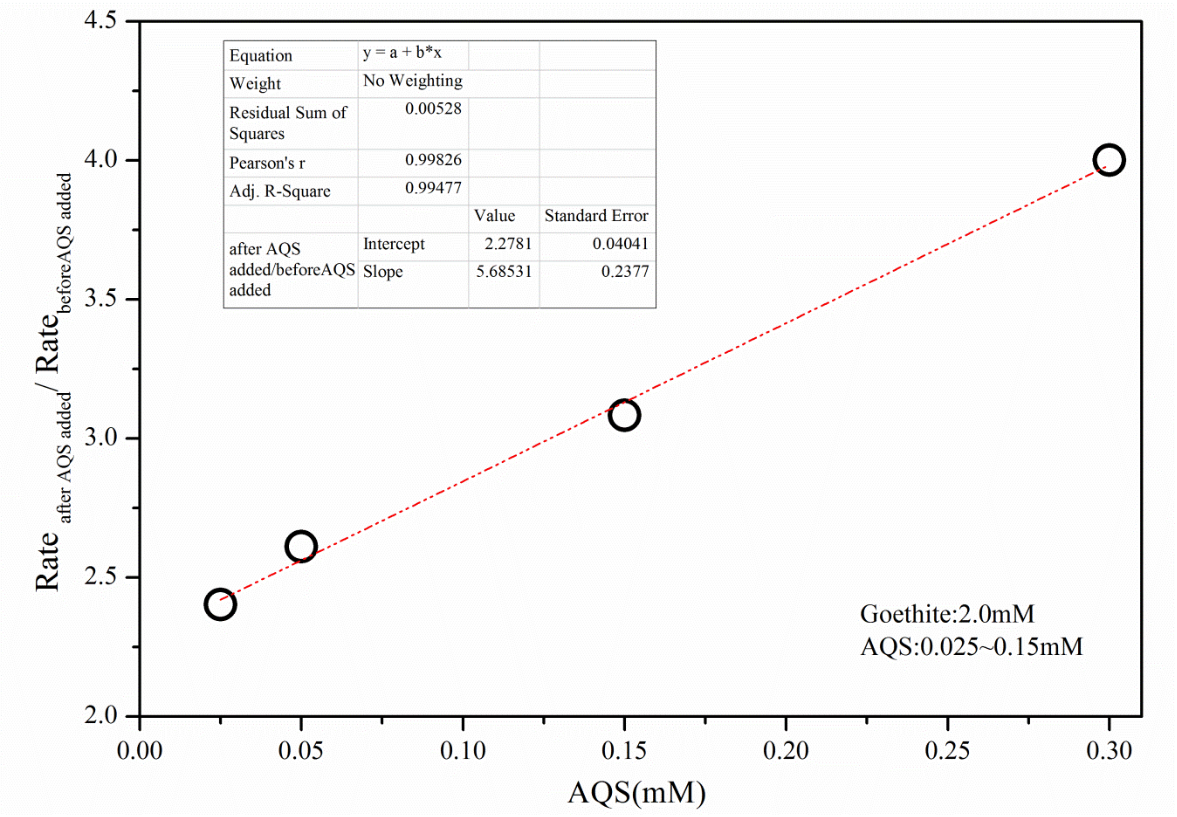

Figure S5 Adsorption capacity of goethite for ferrous iron in “strain S12-goethite” pre-incubation reaction systems containing different concentrations of AQS

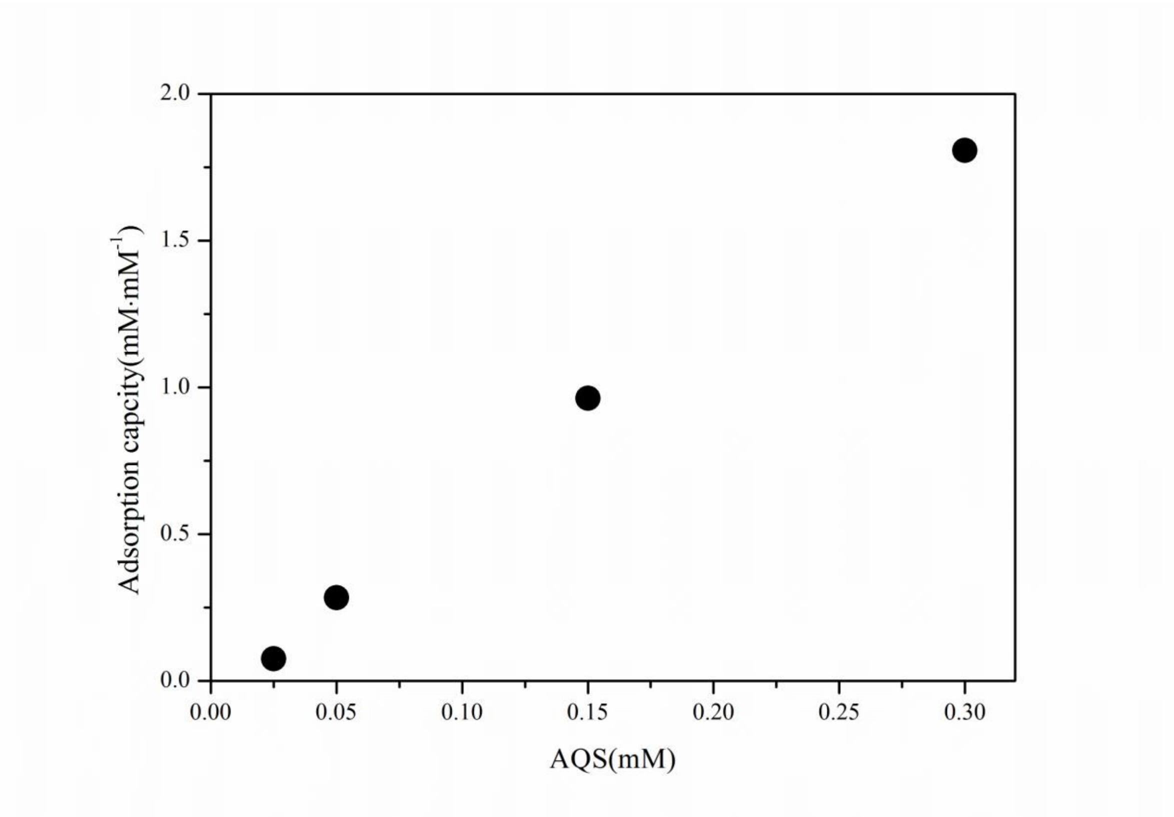

Figure S6 The time course of $Fe(II)_{ads}$ content in "strain S12- AQS" pre-incubation reaction systems added with different concentration of goethite

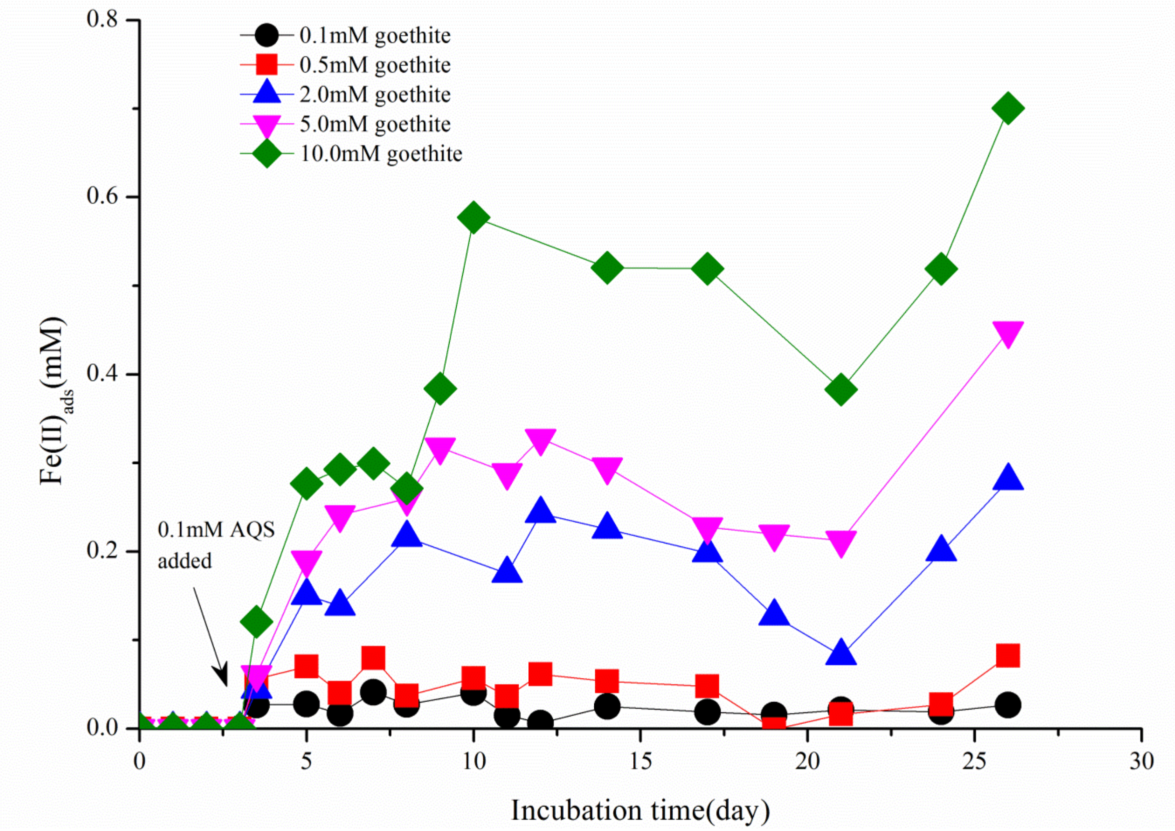

Figure S7 The microbial reduction of AQS_{oxi} to AQS_{red} in "strain S12-goethite" pre-incubation reaction system illustrated by the typical UV/vis spectra of oxidized or reduced forms of AQS during the transformation process

(The UV/vis spectra were acquired in "strain S12- goethite" pre-incubation reaction systems with goethite content at 0.3mM. After 7 days incubation, 0.3 mM AQS was added to reaction systems.)

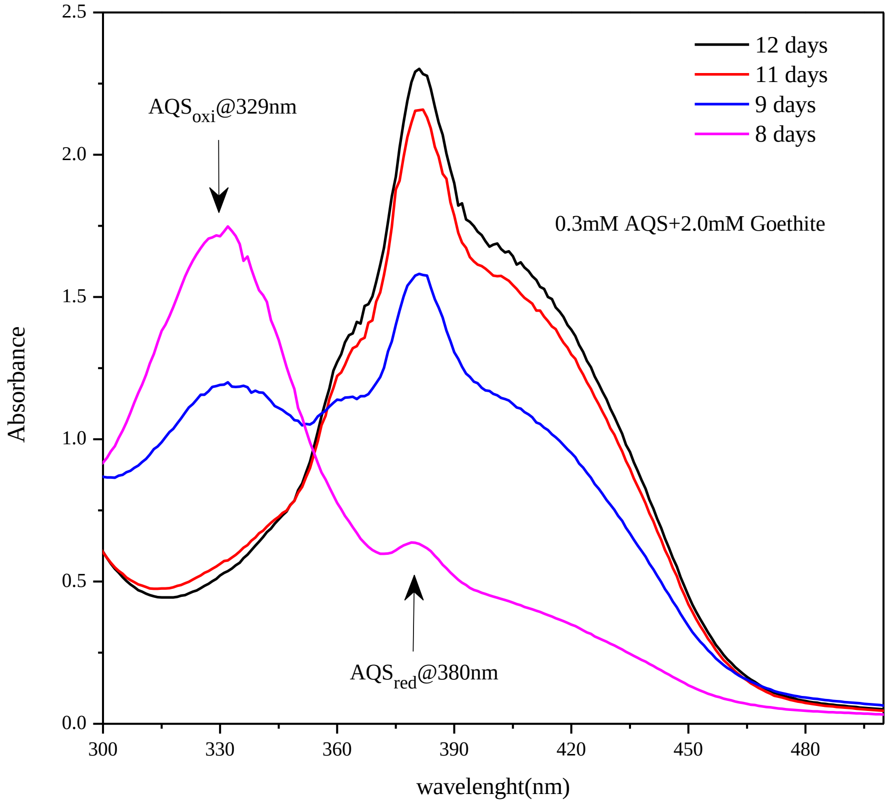

Figure S8 The influence of added goethite contents on the initial AQS_{red} oxidization rate in "strain S12- AQS" pre-incubation reaction systems with low inoculation cell density (5.0×10^7 cells·ml^{-1}).

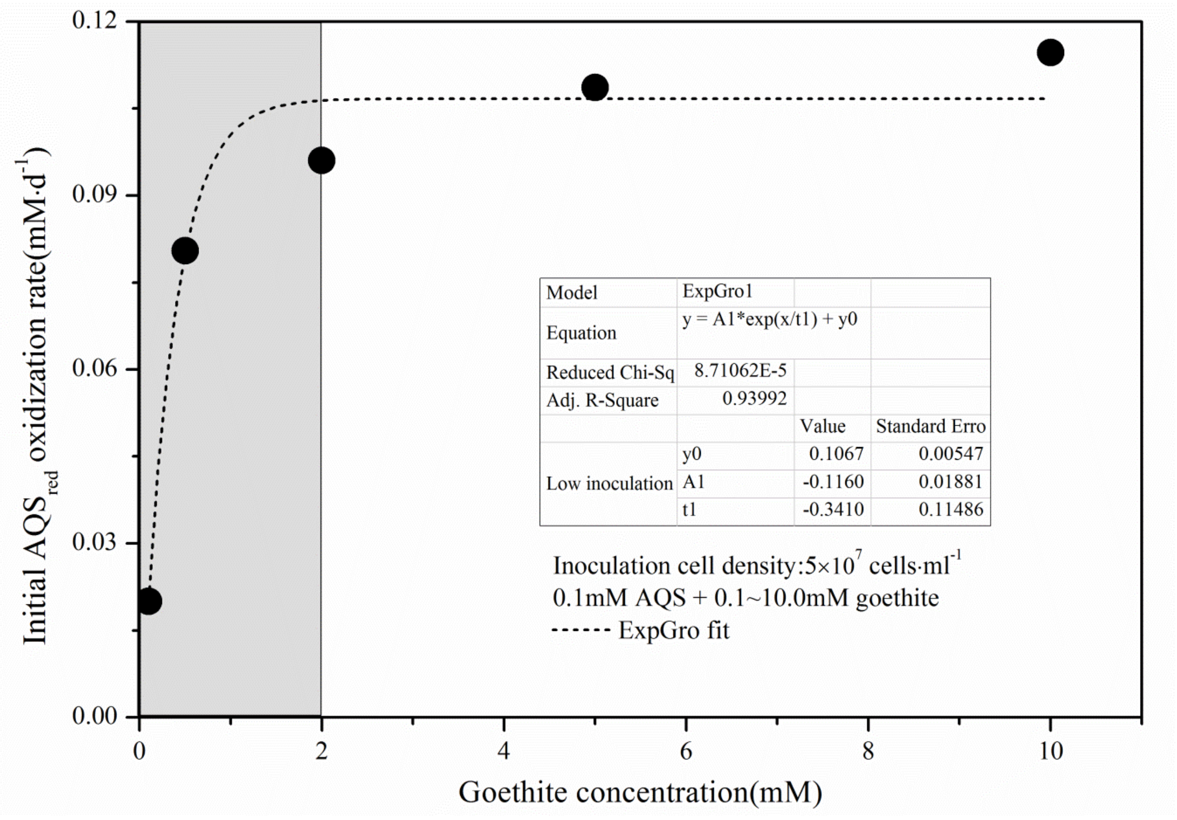

Figure S9 The fitted Monod equation type of initial microbial goethite reduction rates with respected to different inoculation cell density (solid symbol: 2.5×10^{8} cells·ml^{-1}, open symbol: 5.0×10^{7} cells·ml^{-1}, AQS: 0.1mM)

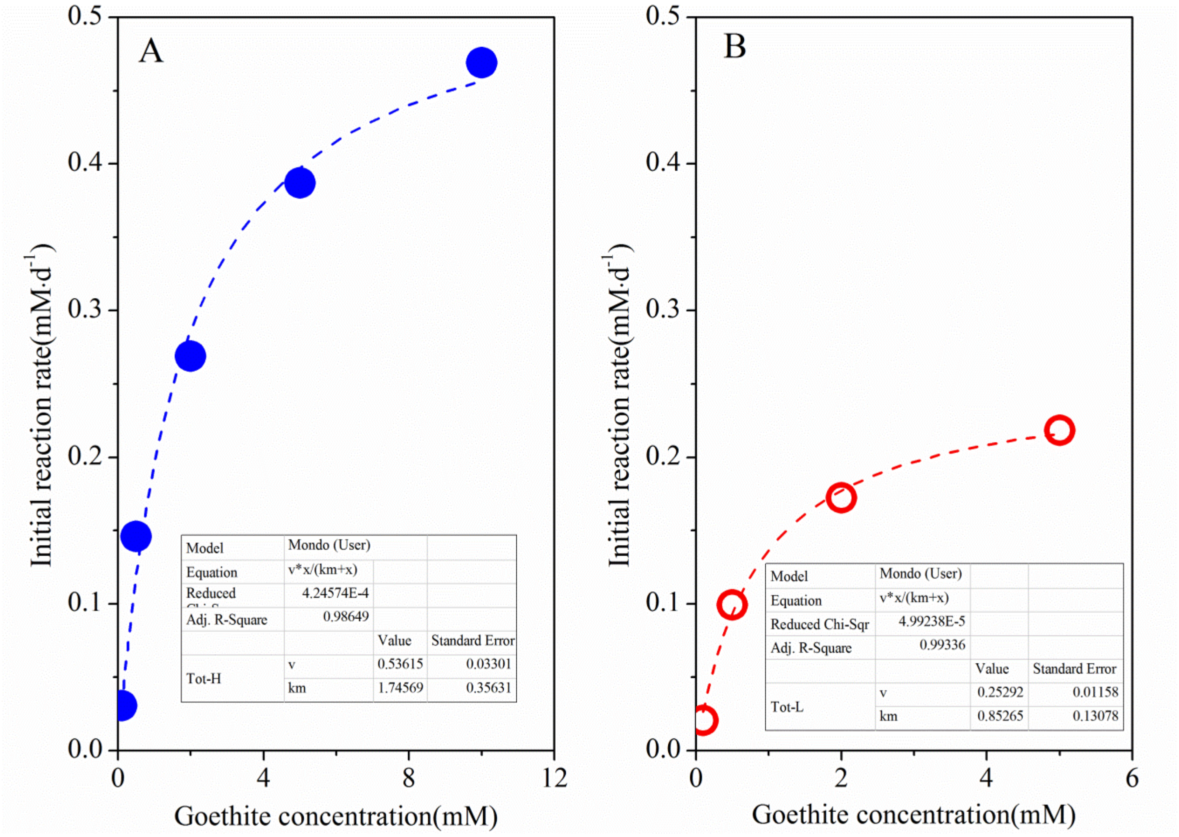

Figure S10 Scanning electron micrograph of prepared goethite

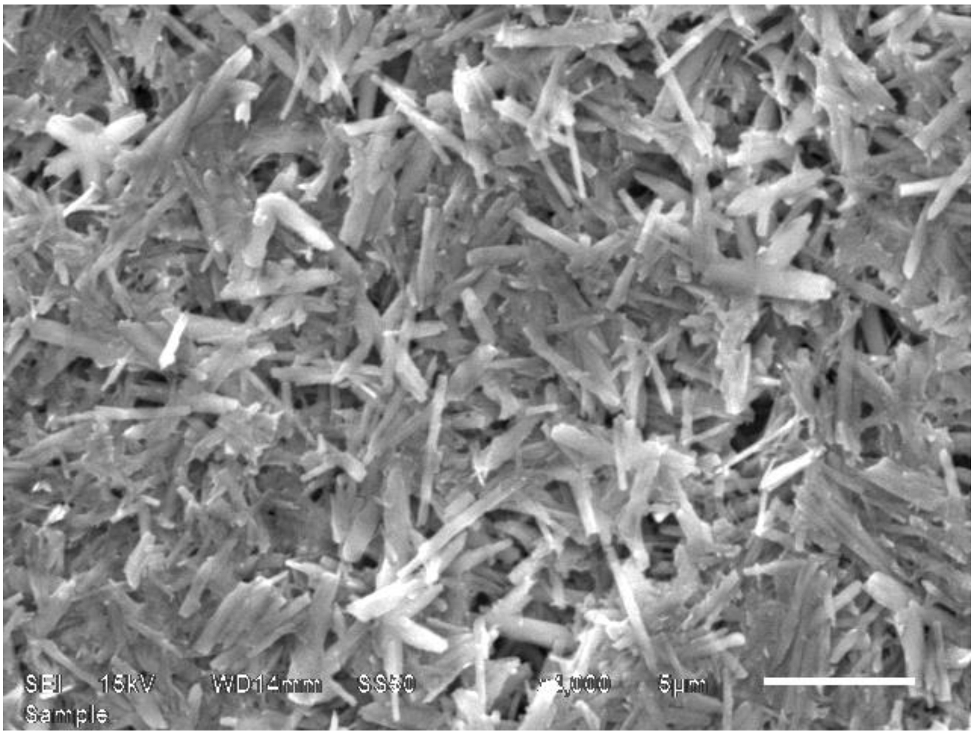

Figure S11 XRD pattern of prepared goethite.

(Red lines: reference pattern PDF No. 29-0713 for goethite)

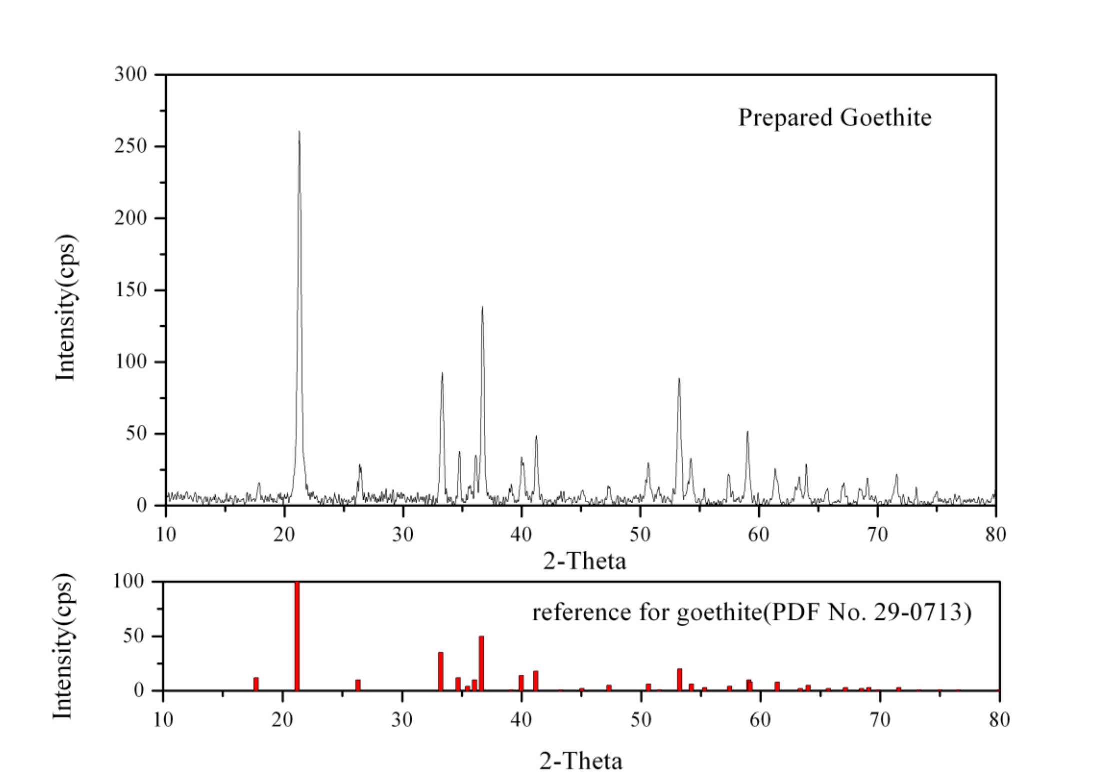

References:

1. Schwertmann, U. & Cornell, R.M. Iron Oxides in the Laboratory: Preparation and Characterization, Edn. 2nd. (Wiley-VCH Verlag GmbH, Weinheim; 2000).
2. Quan, M., Sanchez, D., Wasylkiw, M.F. & Smith, D.K. Voltammetry of Quinones in Unbuffered Aqueous Solution: Reassessing the Roles of Proton Transfer and Hydrogen Bonding in the Aqueous Electrochemistry of Quinones. *Journal of the American Chemical Society* **129**, 12847-12856 (2007).
3. Conant, J.B., Kahn, H.M., Fieser, L.F. & Kurtz, S.S. AN ELECTROCHEMICAL STUDY OF THE REVERSIBLE REDUCTION OF ORGANIC COMPOUNDS1. *Journal of the American Chemical Society* **44**, 1382-1396 (1922).
